# Cross-Species Genome-Wide Identification of Evolutionary Conserved MicroProteins

**DOI:** 10.1093/gbe/evx041

**Published:** 2017-03-01

**Authors:** Daniel Straub, Stephan Wenkel

**Affiliations:** 1 Department of Plant and Environmental Sciences, University of Copenhagen, Frederiksberg C, Denmark; 2 Copenhagen Plant Science Centre, University of Copenhagen, Frederiksberg C, Denmark

**Keywords:** microProteins, miPFinder, protein–protein interaction, metazoa, plants

## Abstract

MicroProteins are small single-domain proteins that act by engaging their targets into different, sometimes nonproductive protein complexes. In order to identify novel microProteins in any sequenced genome of interest, we have developed miPFinder, a program that identifies and classifies potential microProteins. In the past years, several microProteins have been discovered in plants where they are mainly involved in the regulation of development by fine-tuning transcription factor activities. The miPFinder algorithm identifies all up to date known plant microProteins and extends the microProtein concept beyond transcription factors to other protein families. Here, we reveal potential microProtein candidates in several plant and animal reference genomes. A large number of these microProteins are species-specific while others evolved early and are evolutionary highly conserved. Most known microProtein genes originated from large ancestral genes by gene duplication, mutation and subsequent degradation. Gene ontology analysis shows that putative microProtein ancestors are often located in the nucleus, and involved in DNA binding and formation of protein complexes. Additionally, microProtein candidates act in plant transcriptional regulation, signal transduction and anatomical structure development. MiPFinder is freely available to find microProteins in any genome and will aid in the identification of novel microProteins in plants and animals.

## Introduction

Genomes of higher eukaryotic organisms encompass on an average roughly between 15,000 and 25,000 protein-coding genes. Processes such as alternative splicing, alternative promoter usage, alternative polyadenylation and, at the protein level, proteolytic processing, can significantly increase the number of protein variants these organisms can produce. Furthermore, the formation of higher order protein complexes increases the functional diversity of proteins. Such higher order protein complexes are often composed of multiple components. Many proteins also associate with different types of complexes in which they adopt varying roles. MicroProteins have the ability to interfere with larger proteins and hinder them from engaging in higher order protein complexes; they can also sequester their targets into other types of complexes thus providing novel activities. Taken together, microProteins are important and potent modulators of biological processes.

MicroProteins exist as individual transcription units in genomes of higher eukaryotes (*trans*-microProteins) and most of these transcription units evolved during the evolution of genomes where both whole-genome and local duplications and rearrangements resulted in an amplification of protein-coding sequences followed by a subsequent loss of functional domains (Eguen et al. 2015). In addition, alternative transcription processes such as splicing, promoter choice and 3′-end processing can also give rise to mRNA isoforms encoding microProteins (*cis*-microProteins). In either case, the microProtein is related to a larger protein with different functional domains and interferes with the function of these “precursor proteins” (Eguen et al. 2015).

The first characterized protein that qualifies to be referred to as a microProtein, is the helix-loop-helix (HLH) protein INHIBITOR OF DNA-BINDING (ID). ID was identified almost three decades ago (Benezra et al. 1990) as an interaction partner and inhibitor of basic helix-loop-helix (bHLH) transcription factors. The homotypic interaction of ID with a bHLH transcription factor (through the shared helix-loop-helix domain) renders the latter inactive. The first plant microProteins that were discovered are the LITTLE ZIPPERs (ZPR) proteins, which are small proteins containing a single leucine-zipper domain (Wenkel et al. 2007; Kim et al. 2008). ZPR microProteins interact with the much larger class III homeodomain leucine-zipper (HD-ZIPIII) proteins through their leucine-zipper domain and the resulting HD-ZIPIII/ZPR heterodimer is unable to interact with DNA, thus mimicking the ID/bHLH module. In the past years many more microProteins targeting transcription factors have been identified in plants (Eguen et al. 2015). Furthermore, it is possible to design synthetic microProteins that inhibit proteins of interest (Seo et al. 2012). Taken together, these findings indicate that microProtein interference is a powerful way to regulate or fine-tune protein activity.

It is implausible that microProteins are more abundant in plant genomes when compared with animal genomes or that they exclusively target transcription factors. To identify a larger variety of potential microProteins and microProtein regulatory modules in plant and animal genomes, we have performed a computational approach taking protein size, domain organization, known protein interactions and evolutionary origin into account. This approach yielded in the most stringent setting the identification of 1,108 individual high probability microProtein candidates belonging to 482 protein families, with 90 in human, 54 in mouse, 22 in zebrafish, 23 in fruit flies, 36 in *C. elegans*, and 95 in *Arabidopsis*, 204 in tomato, 156 in potato, 94 in rice and 334 in maize. This new microProtein dataset provides a valuable resource for investigating mechanisms of microProtein functions in plants and animals and the miPFinder program can be used to analyze new genomes as soon they become available. As we outline below, miPFinder is tunable and therefore allows relaxation of the stringent setting to identify hidden microProteins for example where protein interactions have not yet been discovered.

## Materials and Methods

### Filtering Incomplete Sequences

Incomplete protein sequences were identified and removed from each protein data set in order to enrich for complete coding sequences. For human and mouse, proteins encoded by the representative protein-coding “GENCODE Basic” transcript set were used. GENCODE combines manual and automatic annotation and aims to annotate all evidence-based gene features in human and mouse genomes at a high accuracy. GENCODE’s Transcript Support Level (TSL) highlights the well-supported and poorly supported transcript models, and transcripts without any transcriptional evidence (TSL5) were omitted. Because the GENCODE annotation is only available for mouse and human, another approach was chosen for the remaining datasets. To deplete incomplete sequences for other organisms, only peptides which were derived from protein-coding nucleotide sequences that contain a start codon (ATG), stop codon (TAA, TGA, TAG), and a length that is a multiple of three were considered.

### Key Features of MicroProtein Candidates

All microProteins known to date are small in size, ranging from 7 to 17 kDa, overall comprising less than 120 amino acids (Eguen et al. 2015). To exert their function, microProteins require only a single functional domain that acts as a protein-interaction platform to sequester their targets. While the sizes of protein domains vary tremendously, the average maximum length of a protein-interaction domain is approximately 100 amino acids (Wheelan et al. 2000). Considering these values and the fact that all known microProteins are less than 120 amino acid in length, we decided to use a maximum length of 140 amino acids to predict novel microProteins.

A second parameter to take into account when trying to identify novel microProtein candidates is the protein organization of potential targets or ancestor. As described earlier, trans-microProteins exist as individual transcription units allowing their evolutionary origin to be traced back. A good example are the plant-specific ZPR proteins that originate from a large homeodomain leucine-zipper ancestor molecule, which got sequentially shortened by gene duplication, degeneration, and truncation (Floyd et al. 2014). The ZPR-ancestor protein is a multi-domain protein that has the ability to homodimerize. In order to predict potential microProteins, we reasoned that a putative microProtein ancestor protein should be large enough to harbor at least two functional domains, consequently we set a minimum ancestor protein size of 250 amino acids. This step also eliminates the identification of small proteins that belong to protein families in which some members are only marginally larger. Finally, we discovered that searches made with a consensus sequence of related microProtein candidates rather than individual protein sequences against a database of larger proteins significantly increases the sensitivity for identifying distantly related sequences, wherefore the microProtein-finder program starts with extracting consensus protein sequences from all small protein families.

### Computational Prediction of Small Related Proteins and Similar Large Sequences

**Fig. 1.—:**
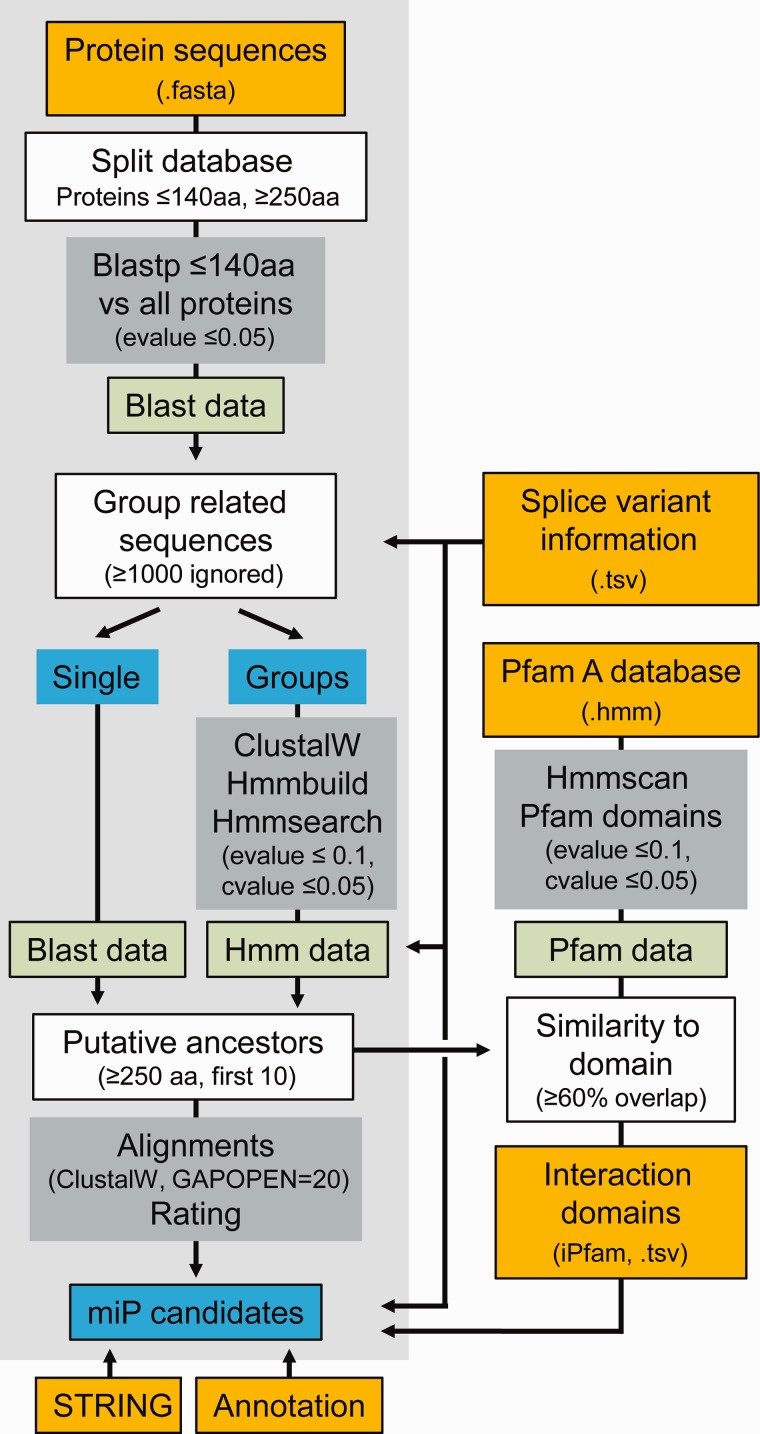
Flow chart miPFinder. Mandatory steps are with a light gray background. Orange, databases; green, data packages; gray, tools; blue, lists; white, custom functions.

In the first step, miPFinder assigns protein sequences as putative microProteins and putative ancestors solely by size (fig. 1). Therefore, the sequence database is divided into small (≤140aa) and large (≥250aa) sequences. Next, BLAST was used to compare all small sequences with each other, resulting in the division of microProteins into single-copy proteins and groups of related sequences (BLAST, cutoff e-value ≤0.001). Each group of small proteins is subsequently aligned (clustalw, gap opening penalty = 20, no end gap separation penalty), combined to a consensus profile (hmmbuild) and compared with all large proteins (hmmsearch, cutoff e-value ≤0.1 and c-Evalue ≤0.05). For ungrouped small sequences (single copy microProteins), similar large proteins are chosen based on the initial BLAST search. Grouped or ungrouped small sequences are considered “microProtein candidate families” and included for further analysis only if they are similar to at least one larger putative ancestor. All putative ancestors are reported in order of significance and up to 10 putative ancestors and their microProtein candidate family are realigned (clustalw, gap opening penalty = 20), rated, and linked in the final report. Additionally, the e-value of the microProtein-ancestor search is stated, which might help in the manual evaluation of microProtein candidates when prioritizing on highly significant similarities.

In addition to the significance values (BLASTP/hmmsearch e-value), we created a rating system that favors known microProteins. This rating is based on the clustalw alignment of the microProtein candidate family and their putative ancestor(s). First, conserved regions (small proteins and ≤10 similar segments of large proteins, BLASTp/hmmsearch) are aligned (clustalw) and regions with low gap content (length ≥20aa and gaps ≤10%) are extracted. This step enriches for regions with high similarity and extracts potential domains. Next, each microProtein candidates and putative ancestors are extracted and two consensus sequences are assembled. The similarity of the consensus sequences is rated based on the Blosum62 table and the following equation:

$$\text{Score}=\sum (\text{log}\;2[(2^{\text{\textasciicircum{}}}\text{Blosum}62)\times \;\text{length}(\text{alignment})\;/\;\text{length}\;(\text{microProtein})])$$

Here, the score is modified by the alignment length in proportion to the length of the microProtein candidate. The resulting alignment rating favors known microProteins and is inversely related to the e-values (supplementary fig. S1, Supplementary Material online), that is a low e-value corresponds to a high microProtein alignment rating.

### MicroProteins Function by Protein Interaction

MicroProtein function requires interaction with respective target proteins. MicroProtein-candidates containing known protein–protein interaction domains, or sequences related to PPI-domains are therefore more likely to function as microProteins compared with small proteins not containing such domains. To identify and annotate protein–protein interaction domains within microProteins and ancestral proteins, miPFinder utilizes the Pfam and iPfam databases.

MiPFinder assigns Pfam domains to all large proteins (hmmscan, cutoff e-value ≤0.1 and c-Evalue ≤0.05), reports domains that have similarity to microProtein candidates (≥60% length of the Pfam domain) in order of significance, and matches these to interchain interaction domains in iPfam. Domains with interchain interaction properties mediate interactions between amino acid chains, a prerequisite for microProtein function.

Additionally, Search Tool for the Retrieval of Interacting Genes/Proteins (STRING) v10 (Szklarczyk et al. 2015) protein-interaction data was retrieved in September 2016 and compared with the list of microProtein candidates. Interaction of a microProtein candidate with a putative ancestor (total score ≥0.4; that is medium confidence) was interpreted as positive indication for microProtein function.

In order to find human microProtein candidates that are associated to diseases, DISEASES v1.0 based on text mining and knowledge was downloaded at http://diseases.jensenlab.org/in December 2015 (Pletscher-Frankild et al. 2015) and employed for information on human microProtein candidates and their putative ancestors.

### Evolutionary Conserved MicroProteins

We employed OrthoFinder v0.3.0 (Emms and Kelly 2015) to uncover homology relationships of microProtein candidates among species. Like other algorithms it performs sequence comparisons via BLAST but additionally normalizes for gene length and phylogenetic distance in cross species comparisons. OrthoFinder outperforms all other commonly used orthogroup inference methods.

Evolutionary conservation of microProtein candidates is visualized using Circos v0.68 (Krzywinski et al. 2009). Only microProtein candidates with similarity to annotated interaction domains (iPfam v1.0, June 2013) were chosen.

For classification of microProtein candidates to Gene Ontology (GO) categories, GO terms for metazoan protein databases were obtained from ENSEMBL and Plant GO terms were retrieved from AgriGO v1.2 (Du et al. 2010). Finally, GOSlimViewer with the generic GOSlim Set from AgBase v2.0 (McCarthy et al. 2006) was used.

Protein classes were assigned to the most significant putative ancestor of each microProtein candidate family using PANTHER v11 (Mi et al. 2017) and collected into higher order classes using protein class relationship information.

### MiPFinder Script, Required Standalone Applications and Database Dependencies

The program is written in python v2.7.9 (Python Software Foundation. Python Language Reference, version 2.7, available at http://www.python.org) and tested for Windows 7. MiPFinder requires the standalone applications hmmer3 (http://hmmer.org/), clustalw2 (http://www.clustal.org/clustal2/) (Larkin et al. 2007) and BLAST2+ obtained from NCBI (ftp://ftp.ncbi.nih.gov/) (Camacho et al. 2009). These applications are freely available and have to be installed separately. Sequence files and databases are not provided, the versions used for the analysis herein are described below. The miPFinder script does not include the filter for full-length mRNA sequences, because the optimal procedure differs between organisms and sequence sources, however, a separate script is available. MiPFinder has been deposited under the GPLv3 license at GitHub (https://github.com/DaStraub/miPFinder).

The interaction domain database iPfam v1.0, June 2013, was obtained from http://www.ipfam.org/(Finn et al. 2014) and Pfam-A_v28.hmm downloaded from Pfam’s FTP site (ftp://ftp.ebi.ac.uk/pub/databases/Pfam/) (Finn et al. 2016). Plant sequence and annotation files were downloaded from Pythozome v11 (http://www.phytozome.net) (Goodstein et al. 2012) (Athaliana_167_TAIR10, Osativa_323_v7.0, Sbicolor_313_v3.1, Slycopersicum_225_iTAGv2.3, Stuberosum_206_v3.4, Zmays_284_5b+). Protein translations of Ensembl 83 gene predictions were acquired from the FTP site for the metazoan datasets and additional information was obtained from Ensembl Genes 83 using biomart (http://www.ensembl.org/biomart) (Caenorhabditis_elegans.WBcel235, Danio_rerio.GRCz10, Drosophila_melanogaster.BDGP6, Homo_sapiens.GRCh38, Mus_musculus.GRCm38).

The miPFinder program takes a single command line in the windows command prompt (e.g. “python miPFinder.py -f proteins.fasta -p ProteinGeneList.tsv -a annotation.tsv”). The minimum input requirement is a simple fasta file with all protein sequences (“-f”), however a file with protein annotations (“-a”) will aid the microProtein selection tremendously. Protein-interaction information from STRING data can be added via “-S” and for the addition of protein–protein-interaction domain information, a Pfam domain database (“-d”) and a file specifying interaction domains (“-i”) is necessary. Moreover, a file specifying the protein–gene relationship (“-p”) will allow for *cis*-microProtein detection, for filtering putative ancestors for their longest splice variant, and for the removal of redundant microProtein candidate splice variants. Parameters for the maximal microProtein and minimum ancestor length can be adjusted (“-M” and “-A”, respectively, standard setting: 140 and 250) as well as all cutoff values.

MiPFinder is built with Python v2.7.9 running on Microsoft Windows 7, and using hmmer v3.1b1, BLAST+ v2.2.29, clustalw v2.1, but any python2, hmmer3, BLAST2, clustalw2 and Microsoft Windows version might be sufficient for running the program. Path to the dependencies (hmmer, BLAST, clustalw) must be specified if the accessory programs are not set as environment variables, using command line arguments “-H”, “-B”, “-C”, respectively. MiPFinder will check the availability of specified input files and correct function of all dependencies before each run.

## Results

### Core Features of MicroProteins

All microProteins known to date are small in size and require only a single functional domain that acts as a protein-interaction platform to sequester their targets. MicroProtein targets are known to be significantly larger than their microProtein counterparts and contain multiple protein domains. The size differences of microProteins and their targets range from 2- to 10-fold, as exemplified by MIP1A/B microProteins and their 3 times larger target CONSTANS (Graeff et al. 2016), and LITTLE ZIPPER microProteins that interact with the 10 times larger class III homeodomain leucine zipper (HD-ZIPIII) proteins (Wenkel et al. 2007). About one-third of all Arabidopsis small proteins are related to larger sequences, which indicates origin by gene duplication, mutation and truncation from larger ancestors as exemplified for LITTLE ZIPPER evolution (Floyd et al. 2014). Additionally, due to their evolutionary origin, microProteins are expected to make up only a fraction of protein families whereas potential ancestors should make up the majority.

The core mechanism of microProtein function relies on their ability to interact with respective target proteins. Protein–protein interactions can be inferred from sequence similarity to an interaction domain or collected from public interaction databases such as STRING. Candidates with indication for interaction capabilities are more likely *bona fide* microProteins than those without such properties, and are therefore preferred.

The miPFinder program takes all these considerations into account (fig. 1) and builds a comprehensive list of microProtein candidates with features that can be interpreted and filtered as required by the individual research question.

### Identifying MicroProtein Candidates with miPFinder

MiPFinder was used to investigate several metazoan and plant genomes with the aim to identify novel microProteins and produce a list of high probability candidates. In most protein databases, sequences are derived from translated RNA transcripts, which in some cases represent only truncated versions of full-length mRNA sequences. In order to prevent these mRNA fragments from being identified as microProtein candidates, human and mouse transcripts without any transcriptional evidence were omitted. For other organisms, only peptides that were derived from transcripts containing a start codon, a stop codon and a length that is a multiple of three were considered. The percentage of sequences that passed the quality filter varied considerably. In most organisms, >98% protein sequences appeared to be complete, however in maize and zebrafish only 91% and 72% of the protein sequences passed the filter. Additionally, 60% of human and 72% of mouse transcripts and their corresponding proteins are in Ensembls GENCODE basic set, and of these, ∼80% are either with transcriptional evidence or not tested for expression (table 1).

**Table 1:** Overview of miPFinder Results

Species	Protein sequences			microProtein candidate families								
	Original	Filtered^a^	%	Total	Trans-miP^b^	%^c^	PPID^d^	%^c^	PPI^e^	%^c^	≥50% ≥250aa^f^	%^c^
*Arabidopsis thaliana*	35386	35364	99.94	551	531	96	193	35	80	15	328	60
*Solanum lycopersicon*	34727	34415	99.10	1767	1767	100	419	24	140	8	1344	76
*Solanum tuberosum*	51472	50631	98.37	2772	1422	51	587	21	106	4	2011	73
*Sorghum bicolor*	47205	46544	98.60	866	861	99	206	24	n.d.	n.d.	557	64
*Oryza sativa*	52424	52417	99.99	1661	1578	95	305	18	62	4	1090	66
*Zea mays*	88760	80694	90.91	5132	3673	72	1007	20	195	4	3688	72
*Homo sapiens*	101933	48542	47.62	1235	320	26	340	28	44	4	850	69
*Mus musculus*	56337	31983	56.77	526	221	42	186	35	48	9	346	66
*Danio rerio*	44487	32031	72.00	371	201	54	165	44	28	8	253	68
*Drosophila melanogaster*	30362	30152	99.31	218	128	59	74	34	24	11	124	57
*Caenorhapditis elegans*	30939	30925	99.95	768	372	48	168	22	41	5	551	72

a Coding sequence length is a multiple of 3 and contains a start and a stop codon; for *H. sapiens* and *M. Musculus* protein coding sequences of GENCODE basic that are not flagged as lacking any transcription evidence.

b MicroProtein candidates do not contain a cis-miP.

c Percentage of total microProtein candidate families (column “Total”).

d Sequences with annotated protein–protein interaction domain (PPID).

e Protein–protein interaction of at least one microProtein candidate with at least one putative ancestor according to STRING data.

f ≥50% of related sequences are ≥250aa in length.

n.d., not determined.

Following the enrichment of full-length sequences, the respective datasets were analyzed with miPFinder. The resulting microProtein candidates are annotated with various information, such as whether they are alternative gene products, similar to an interaction domain, known to interact with one of their potential ancestors, and the size distribution of related sequences to allow filtering for specific features and to enrich for high probability candidates (supplementary table S1, Supplementary Material online).

In plants, groups without *cis*-microProtein candidates, which are alternative products of their ancestor genes, make up the majority of microProteins identified in these species, although in potato and maize these numbers are considerably lower (51% and 72%, respectively, see table 1). In metazoans, small splice variants of large proteins are present in more than half of the microProtein candidate families. For example, only 26% of human candidate microProtein families are exclusively composed of *trans*-microProteins. The number of splice variants per gene, which is significantly higher in mammals than in plants, might explain these differences (Kim et al. 2007). However, invertebrates and plants have a similar proportion of spliced genes (Kim et al. 2007), and the difference in this situation might be due to the dissimilar annotation degree of splice variants among the databases.

In Arabidopsis, ∼35% of microProtein candidate families have similarities to known protein–protein interaction domains of putative ancestral proteins and 15% have at least one microProtein candidate that interacts with a putative ancestor, indicating the possibility of microProtein function. When looking at all species, most microProtein candidates are in protein families where larger proteins represent the majority of the protein family; this is similar to what is observed in known microProtein families.

In summary, we define high probability microProtein candidates as small proteins that are known to interact with related large potential ancestor(s) and are part of protein families where the larger proteins represent the majority of the respective protein family. This set of high probability microProtein candidates was further used to validate the method and to identify novel microProteins.

### Detection of Known MicroProteins Using miPFinder

In order to validate and test our computational approach, we employed miPFinder on the Arabidopsis genome and found that 18 of the 22 known Arabidopsis microProteins (table 2) are present in the list of high probability microProtein candidates. LITTLE ZIPPER (ZPR) (Wenkel et al. 2007) and MIP1A/MIP1B (Graeff et al. 2016) are exclusively grouped according to their microProtein family associations, indicating that miPFinder is also able to cluster sequences correctly. MYB-microProteins (Tominaga-Wada et al. 2011) and HLH-microProteins (Wang et al. 2009; Zhang et al. 2009) families harbor additional members that have not been studied to date, but these proteins are likely microProteins with similar function. HLH-microProteins are divided into KDR-ILI1-like and PAR-like subgroups because of specific sequence differences. MYB-microProteins, HLH-microProtein, and MIP1A/MIP1B are correctly reported as being similar to an interaction domain, whereas ZPR’s domain bZIP-TF is not annotated as interaction domain. MINI ZINC FINGER (Hu and Ma 2006) are not in the set of high probability candidates because they have a low proportion of large protein ancestors. KNATM on the other hand is not reported to interact with any of its potential ancestors thus making it not a high probability candidate. MiPFinder retains all these microProteins irrespective of their interaction characteristics or size proportions and reports their features; therefore the user can decide whether to rely on these restrictions or not. Since miPFinder performs very well in the recall of known microProteins in Arabidopsis, we were interested in finding potential microProteins that are relevant to human health.

**Table 2:** Known MicroProteins Identified by miPFinder

MicroProtein group members^a^	Ancestor count	Known miPs	Rating	Min. evalue	cis-mip^b^	% small^c^	% medium^d^	% large^e^	Pfam^f^	PPID^g^	PPI^h^	
AT2G45450.1; AT3G60890.1; *AT3G52770.1*	4	ZPR3	147	3.9E-06	no	50	0	50	bZIP transcription factor	Yes	Yes	
*AT4G01060.1*; *AT2G46410.1*; AT1G43330.1; *AT2G30432.1*; AT2G13960.2; *AT2G30420.1*; AT1G66380.1; *AT5G53200.1*; *AT2G30424.1*; *AT1G01380.1*	125	TCL1, TCL2, ETC1, ETC2, CPC, ETC3, TRY	223	9.5E-27	no	8	16	76	Myb-like DNA-binding domain	Yes	Yes	
*AT2G42870.1*; AT2G47270.1; *AT3G58850.1*	56	PAR1, PAR2	182	1.3E-09	no	9	25	66	Helix-loop-helix DNA-binding domain	Yes	Yes	
*AT5G39860.1*; *AT1G26945.1*; *AT5G15160.1*; *AT3G28857.1*; *AT1G74500.1*; *AT3G47710.1*	10	PRE3, PRE5, BNQ3, KDR, BNQ2, PRE1	147	9.3E-06	no	33	11	56	Helix-loop-helix DNA-binding domain	Yes	Yes	
*AT4G15248.1*; *AT3G21890.1*	22	MIP1A, MIP1B	229	2.0E-15	no	6	29	65	B-box zinc finger	Yes	Yes	
*AT3G28917.1*; *AT1G74660.1*; *AT1G18835.1*	8	MIF1, MIF2, MIF3	288	1.0E-31	no	18	35	47	ZF-HD protein dimerization region	No	Yes	
*AT1G14760.2*	8	KNATM	174	5.0E-17	no	17	17	67	KNOX2 domain	No	No	

a Only one protein identifier per gene is shown. Gene identifiers of known microProteins are in italics.

b Whether microProtein candidates contain cis-miPs.

c Percent of related sequences (BLAST or hmmsearch) that are ≤140aa in length.

d Percent of related sequences (BLAST or hmmsearch) that are 141–249aa in length.

e Percent of related sequences (BLAST or hmmsearch) that are ≥250aa in length.

f pfam domain of highest score.

g Whether pfam domain is annotated as protein–protein interaction domain.

h Protein–protein interaction of at least one microProtein with at least one related large sequence according to STRING database.

### Disease-Related MicroProteins in Human

Because microProteins act as dominant regulators of protein function, it is conceivable that they underlie diseases when mutated. It is conceivable that mutations in microProteins that are involved in the regulation of basic cell development or cell proliferation might cause normal cells to develop into cancer cells. We found that ∼10% of all small proteins encoded in the human genome are represented in the DISEASES database, a text mining database for disease-associated proteins (Pletscher-Frankild et al. 2015). The majority (60%, significant enrichment, Fisher’s Exact Test *P* value < 0.01) of all human high confidence microProtein candidates are disease-associated, and around one-third is associated with severe diseases such as cancer (supplementary fig. S2, Supplementary Material online). This high percentage of disease-related microProtein candidates emphasizes the potential importance of miPFinder results. MicroProteins could be a yet overseen cause for diseases and discoveries of disease-associated microProteins might open new avenues for cures in the futures. To further show the validity of disease associated microProtein candidates identified by miPFinder we describe two small proteins with probable microProtein function below.

### ALT-PTK6 and POP2, Two Examples of Well-Studied Human MicroProtein Candidates in Disease

Among high probability microProtein candidates identified by miPFinder are two well-studied examples in human: POP2 and ALT-PTK6. The 97 amino acids PYD-only protein 2 (POP2) is a high probability microProtein candidate that interacts with NLR family proteins that are part of inflammasome complexes and thereby disrupt inflammasome assembly (Dorfleutner et al. 2007). POP2 also modulates NF-κB (Bedoya et al. 2007), a key regulator of immune reaction that has been linked to cancer. Furthermore, POP2 is one of four similar small proteins in human that all interfere with essential PYD–PYD interactions (Chu et al. 2015). POP2 is a credible microProtein that regulates nontranscription factors.

Protein tyrosine kinase 6 (PTK6), also called breast tumor kinase (BRK), promotes in disease oncogenic signaling possibly due to intracellular localization (Brauer and Tyner 2010). The *PTK6* gene produces two splice variants, the 52-kDa full length PTK6 protein and a 15-kDa alternative splice product, named ALT-PTK6, which miPFinder discovered as potential microProtein. Even though ALT-PTK6 and full length PTK6 interaction is not detectable, ALT-PTK6 associates with PTK6 substrates and coexpression of both PTK6 and ALT-PTK6 negatively modulates PTK6 protein–protein associations, possibly by competitive binding (Brauer et al. 2011).

These two examples showcase the potential of miPFinder results and its implication in human health. Both examples seem to fit the microProtein mode of action and act at important hubs for human well-being.

### Evolutionary Conserved MicroProteins

To this end, we identified high probability microProtein candidates that are known to interact with their potential targets and we describe one example of one of these top‐ranked candidates that can be linked to evidence that strongly points to microProtein function. The formation of protein complexes is a prerequisite for microProtein function and using the protein–protein interaction database STRING to define high probability microProtein candidates proved valuable on the one hand but is also very restrictive on the other. Using the STRING database to test the interaction between a microProtein candidate and its potential ancestor(s) disregards common interactions with a third protein, thus limiting detectable associations to only homotypic domain interactions. An alternative less restrictive approach is to filter for sequence conservation. Because proteins that are conserved in several related species are more likely to have retained a function under evolutionary pressure. Additionally, conserved sequences are less prone to be annotation artifacts or degenerated pseudo-genes. However, it is important to note, that species-specific microProteins should not be ignored because they could be involved in species-specific traits and in some cases might even have acted as facilitators of speciation.

To assign evolutionary conservation to microProteins, individual microProtein candidates were combined with OrthoFinder (Emms and Kelly 2015) results. The OrthoFinder algorithm identifies homology relationships between sequences while solving biases in whole genome comparisons and is therefore more accurate than other orthogroup inference methods.

Individual microProtein candidates were tested for their presence in all 11 species that were examined in this study. The known microProteins ZPR, MIF, Myb-microProteins, and HLH-microProtein (KDR-ILI1-like subgroup) are identified in all plants, while the HLH-type microProteins (PAR subgroup) and MIP1A/MIP1B are only present in dicotyledonous plants.

**Fig. 2.—:**
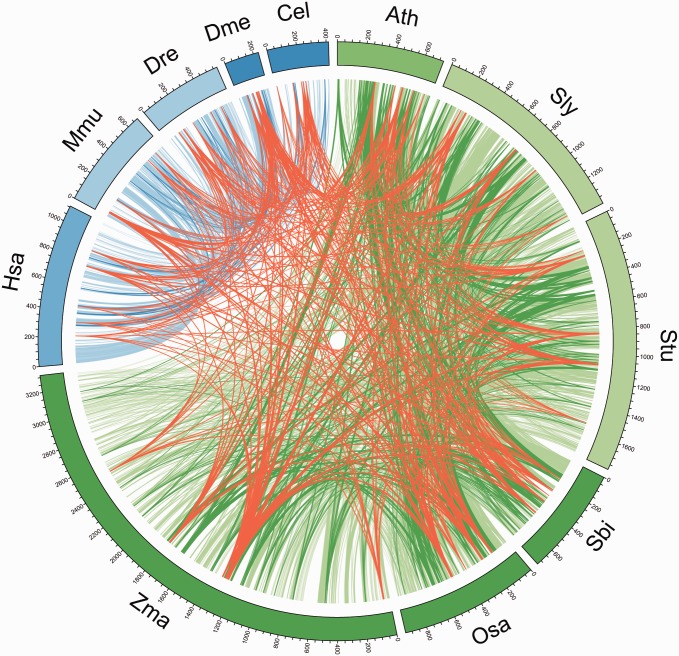
Circos plot of individual microProtein candidates. Links indicate conservation between species based on OrthoFinder. Red, in all 11 species; dark blue, exclusively in all five metazoans; light blue, only in metazoans; dark green, exclusively in all six plants; light green, only in plants.

Using this evolutionary conservation approach, we identified microProteins that are exclusively found either in plants or metazoans. Each plant dataset contains several hundred to thousands of microProtein candidates that are exclusively conserved among plants. Around 100 proteins in approximately 30 microProtein candidate families per plant have related sequences in all other plants (fig. 2, dark green and table 3). One-third of these are DNA-binding or transcription factor-related domains, such as MYB, helix-loop-helix, or zinc finger. A larger number of microProtein candidates, ranging from 444 in *Sorghum bicolor* to 2,055 in maize, are conserved in at least two plant species (fig. 2, light green and table 3).

**Table 3:** Conserved MicroProtein Candidates

Species	miP candidates				Excl. in metazoa^a^		Excl. in all metazoa		Excl. in plants^a^		Excl. in all plants		In all species		Total PRT	%^b^
	Total	%^c^	PPI^d^	%^b^	PRT	GRP	PRT	GRP	PRT	GRP	PRT	GRP	PRT	GRP		
*Arabidopsis thaliana*	1589	5	751	47					461	151	105	30	22	7	588	37
*Solanum lycopersicon*	4160	12	1399	34					1554	641	108	31	24	7	1686	41
*Solanum tuberosum*	6215	12	1784	29					1874	902	116	32	21	7	2011	32
*Sorghum bicolor*	1990	4	733	37					444	165	87	30	17	7	548	28
*Oryza sativa*	3447	7	945	27					678	299	103	30	29	7	810	23
*Zea mays*	10591	13	3315	31					2055	955	119	33	33	7	2207	21
*Homo sapiens*	2841	6	1107	39	530	161	14	3					15	7	559	20
*Mus musculus*	1209	4	681	56	358	132	8	4					16	7	382	32
*Danio rerio*	907	3	576	64	203	81	5	3					14	7	222	24
*Drosophila melanogaster*	567	2	235	41	73	24	8	3					22	7	103	18
*Caenorhapditis elegans*	1324	4	416	31	58	41	6	3					11	7	75	6
total	34840		11942		1222	439	41	16	7066	3113	638	186	224	77	9191	

a Exclusively in the specified group but not conserved among all.

b Percentage of total microProtein candidate sequences (column “Total”).

c Percentage of filtered sequences (table 2, column “Filtered”).

d Sequences with annotated protein–protein interaction domain.

PRT, number of protein sequences; GRP, number of miP candidate families (groups).

In metazoans, 10 microProtein candidates are conserved in all analyzed genomes (fig. 2, dark blue and table 3). These sequences have similarity to three structures, the nuclear transport factor 2 (NTF2) domain, ankyrin repeats, and the PDZ domain. The NTF2 families consist in majority of small proteins in contrast to the other two families, which have less than one-tenth of small protein sequences and are therefore preferred microProtein candidates. These numbers differ considerably from microProteins in plants, which might be caused by a bigger evolutionary distance between the chosen metazoan genomes than between the relative closely related plant genomes. Some dozen to hundreds (from *C. elegans* with 58–530 in human) of proteins are conserved exclusively among at least two of the five metazoan proteomes (fig. 2, light blue and table 3). The number of human miP candidates that are conserved in at least two of the investigated animal genomes (530) is comparable to the corresponding number in Arabidopsis (461), therefore the incidence of microProteins might be similar in animals and plants.

**Fig. 3.—:**
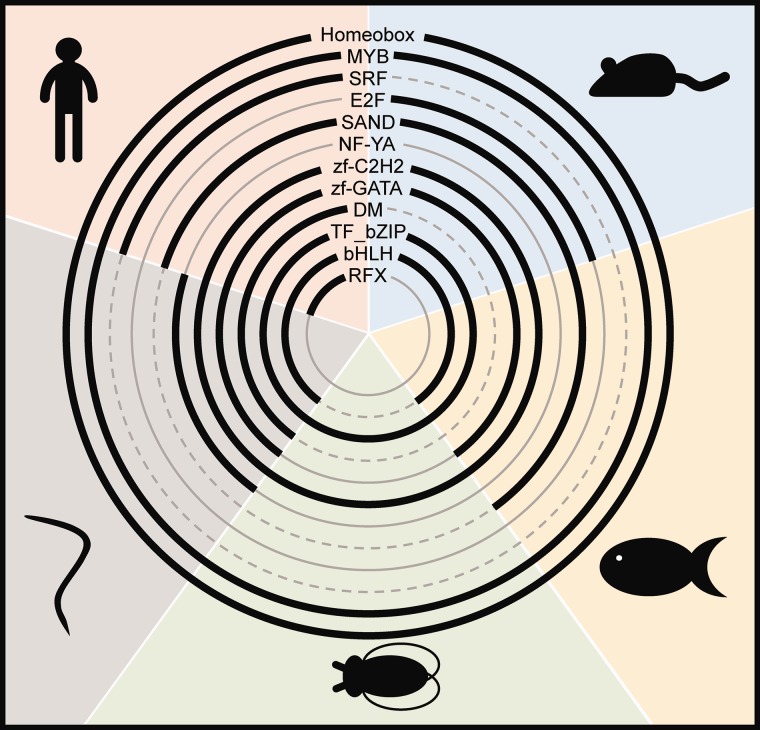
MicroProtein candidates in transcription factor families in metazoans. The presence of microProtein candidates in human (upper left, red), mouse (upper right, blue), zebrafish (right, yellow), fruit fly (bottom, green) and roundworm (left, gray) in the respective transcription factor family is indicated as bold line.

Metazoan microProtein candidates and their putative ancestors were classified into six transcription factor groups and 70 families according to AnimalTFDB (Zhang et al. 2015). Around 10% of microProtein candidate families (Human 117, mouse 54, zebrafish 40, *D. melanogaster* 17, *C. elegans* 43) contained at least one transcription factor (TF). TF Basic Domains Group (e.g. bZIP), Helix-turn-helix (e.g. MYB, homeobox), Other Alpha-Helix Group and Zinc-Coordinating Group (e.g. zf-C2H2) have microProtein candidates in all investigated metazoans (fig. 3). Some TF families with microProtein candidates were present in several species (e.g. SAND, DM, bHLH, zf-GATA) and few were species specific (e.g. SRF and RFX in human, E2F in mouse, NF-YA in *C. elegans*) (fig. 3).

### Gene Ontology Analysis of MicroProtein Candidates

**Fig. 4.—:**
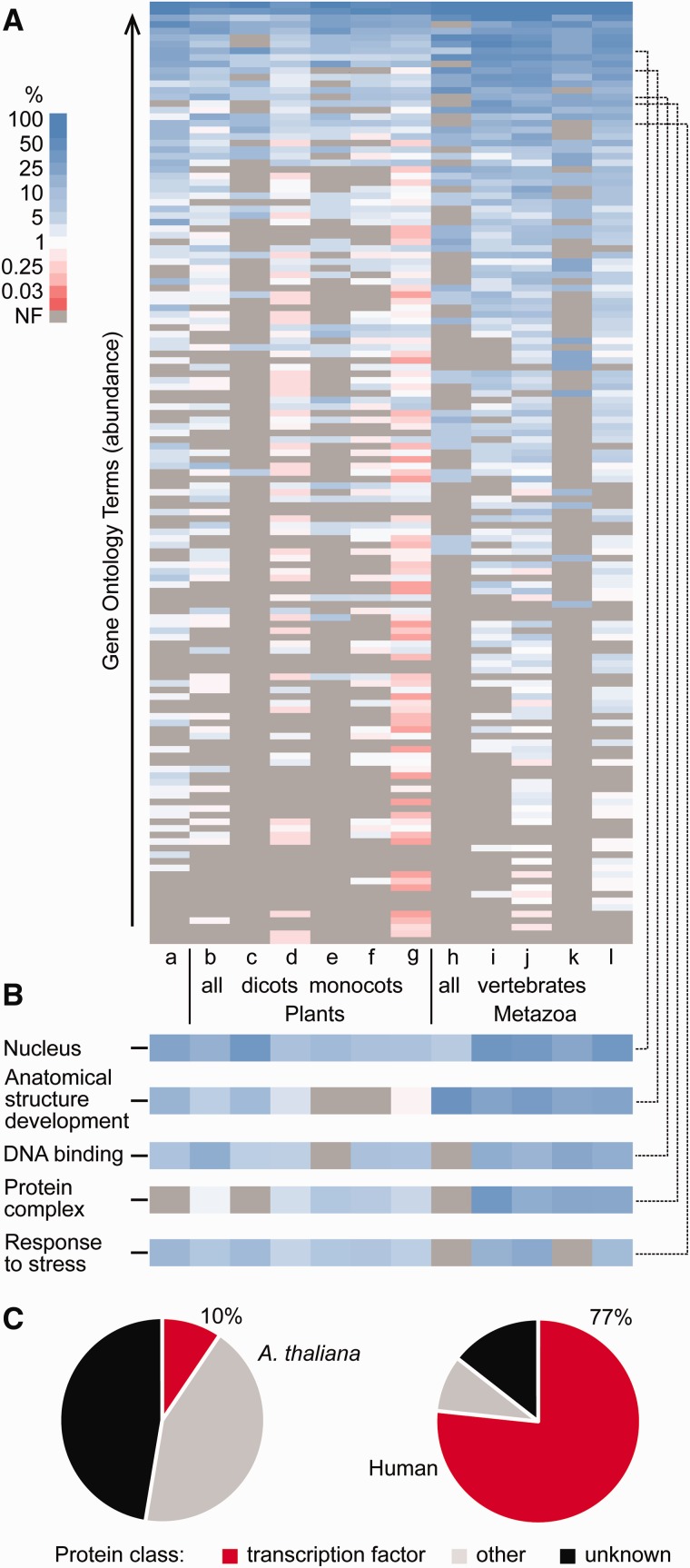
Gene Ontology and protein class analysis of microProtein-subsets. For all sets, only the most significant ancestor of a microProtein candidate family was analyzed. (*A* and *B*) The subsets represent microProtein candidate families with the following conservation in: a: all species; b: all plants; c: all dicots; d: some dicots; e: all monocots; f: some monocots; g: some plants; h: all metazoa; i: all vertebrates; j: some vertebrates; k: nonvertebrates; l: some metazoa. (*A*) The GO terms are sorted in order of their descending average abundance of all subsets and color coded by their subset specific percent of genes with GO annotation. (*B*) Selected GO terms extracted from A as indicated by dashed lines. NF, not found. (*C*) Protein classes that are regulated by Arabidopsis (left) and human (right) high probability microProteins.

Gene Ontology (GO) terms describe gene products in terms of their associated biological processes, cellular components and molecular functions in a species-independent manner. In order to visualize and summarize the function of microProtein candidates, the most significant ancestor of each microProtein candidate family was analyzed for its GO annotation. MicroProtein candidate families were divided into several subsets based on their conservation according to OrthoFinder to investigate their roles in different evolutionary backgrounds (fig. 4). According to GO classifications, many microProtein ancestors are located in the nucleus throughout the subsets, and are involved in DNA binding and in protein complexes. Since all known microProteins are regulating transcription factors by altering complex formation, a notable proportion of these features is expected and was identified. The biological process “Anatomical Structure Development” is mostly annotated for metazoan proteins, but also present in dicots but not in monocots. In plant and some metazoan subsets many proteins are involved in response to stress (fig. 4*B*). These results support the function of the ancestral genes of known microProteins, which are involved in signal transduction, stress responses, and development.

### Protein Classes Regulated by MicroProteins

PANTHER annotates proteins through evolutionary relationship with descriptive protein classes (Mi et al. 2017). According to this classification, in Arabidopsis only 10% of high probability microProtein candidates putatively regulate transcription factors while in human it is as much as 77% (fig. 4*C*). These numbers are approximately halved when considering the set of evolutionary conserved microProtein candidates, but this is largely due to the increase in the proportion of unknown classification (supplementary fig. S3, Supplementary Material online). Besides regulating transcription factors, high probability microProteins in Arabidopsis target enzymes like hydrolases (5%), oxidoreductases (4%) and ligases (10%). Among human conserved microProteins are many involved with signaling molecules (13%) and enzyme modulators (7%). Taken together, microProtein candidates regulating transcription factors are by far in the majority in human but not in Arabidopsis but more importantly it seems that the microProtein-regulation extends beyond transcription factors.

## Discussion

We have developed miPFinder, a program that both identifies and classifies microProteins, which are important regulators of protein function. MiPFinder starts with a set of protein sequences and considers information about protein size, sequence similarity, domain composition and protein interaction to create a list of microProtein candidates. Additionally, when combined with protein conservation information, miPFinder can discriminate between microProteins that occur in several species or microProteins that are species-specific. This resource will aid the identification of microProteins and will promote research on the function of novel microProteins.

An earlier version of miPFinder identified the Arabidopsis microProteins MIP1A and MIP1B (Graeff et al. 2016) that control flowering by recruiting a known flowering activator into a repressive complex. This earlier version was used to identify transcription factor-related microProteins, this new version extends the microProtein concept to any class of protein.

Because microProteins are under-investigated in animals compared with plants, we aimed to find microProtein candidates related to human health among miPFinder results. As our results suggest, microProteins might play important roles in disease development and mutations in microProtein encoding genes could deregulate vital systems like cell cycle regulation that might eventually lead to cancer. A large percentage of human high probability microProtein candidates are disease related further supporting this notion. A particularly interesting case is the alternative splice variant of breast tumor kinase (BRK or PTK6). The human PTK6 has strong indications for microProtein function, such as coexpression of full length PTK6 and the alternative product (ALT-PTK6) negatively modulated PTK6 protein–protein associations and ALT-PTK6 seemingly competed with the full-length protein for interaction partners (Brauer et al. 2011). This compelling example shows the potential of miPFinder results that might represent only the tip of the iceberg.

### Limitations of MicroProtein Identification

Because microProteins act by engaging in direct protein–protein interactions, candidates with similarity to a known protein-interaction domain are more promising than those without any known domains. MiPFinder annotates protein domains to a given set of sequences, but already existing domain information can also be provided if desired. However, some proteins interact via discontinuous sequences that form three-dimensional interaction interphases rather than with specific interaction domains. Databases such as STRING contain known protein–protein interactions independent of domain annotations and infer these to evolutionary conserved proteins in different species, even so, only a fraction of in vivo interactions might be captured. Due to these constraints, miPFinder does not filter for interaction abilities it simply annotates potential common interaction domains and known protein–protein interactions of microProteins and their related larger target proteins. Thus highest priority can be given to microProtein candidates with known interactions or interaction abilities but the search also includes all other candidates.

Using miPFinder, we screened metazoan and plant genomes for microProteins and found that all 22 known Arabidopsis microProteins were identified. About 18 of these 22 are among the high probability candidates that resemble microProtein candidates that are annotated to interact with their putative ancestor and are in protein families where larger proteins represent the majority of the protein family. The first identified microProtein, Inhibitor of DNA binding (ID), was initially identified in mice (Benezra et al. 1990) and miPFinder is able to identify ID2 and ID3 in mouse, however ID1 and ID4 are omitted due to the arbitrary size restriction of miPFinder to proteins smaller than 140 amino acids (ID and ID4 are 148aa and 161aa in size). MicroProteins are not always encoded as individual transcription units (*trans*-microProteins) as seen in the case of *cis*-microProteins which are splice variants of larger proteins. The human *cis*-microProtein of Regulator of G-protein signaling 5 (RGS5), a small splice variant that can negatively inhibit its targets function (Liang et al. 2005), is not identified by miPFinder, because the supposedly large ancestor RGS5 is shorter (201aa) than miPFinder standard setting allows (≥250aa). To allow for adjustments in microProtein candidate detection, the parameters for the maximum microProtein and minimum ancestor length are easily changeable in miPFinder.

### Evolutionary Conserved MicroProteins

Focusing solely on microProtein candidates with annotated protein–protein interactions with their putative ancestors proved valuable in finding high confidence microProteins but was also very restrictive enriching for well-studied proteins. An alternative approach considers conservation information in order to enrich for proteins with function under evolutionary pressure. This approach yields much more diverse microProtein candidates but lacks the confidence for protein–protein interaction.

Several known Arabidopsis microProteins can be found in either all of the six plant genomes that we have investigated here or in at least in one of the subsets of the three dicot or monocot genomes. MicroProtein candidates that are conserved among all investigated species (plants and animals) seem less likely to have microProtein function because related sequences of these proteins are overall relatively small and larger protein sequences are only distantly similar. In general, we find that microProteins that are conserved in at least a few other species have an increased probability that the small, often one exon sized microProtein candidates are not pseudogenes. Consequently, microProtein candidate families that are conserved in several but not all of the 11 genomes are promising candidates. Good examples are the LITTLE ZIPPER microProteins, which regulate leaf development and that are conserved in the whole plant euphyllophyte clade, and MIP1A/B, which have been shown to fine-tune flowering of Arabidopsis, which are conserved in all dicotyledonous plants.

To learn more about the biological processes both microProteins and their putative targets are involved in, we categorized the most significant ancestor of each microProtein candidate family into functional groups and performed a Gene Ontology (GO) analysis. MiPFinder results showed high percentage of GO terms that are also found among ancestors of known microProteins such as “signal transduction” and “anatomical structure development” including several microProtein candidates that are related to transcription factors. This underlines the importance of microProteins in mediating responses to the environment and basic patterning pathways, which are exemplified in the role of known microProteins, such as ZPRs in Arabidopsis leaf development.

Since known plant microProteins are involved in regulation of transcription, we compared our metazoan miPFinder results to a transcription factor database. Putative microProteins are present in several major transcription factor families in all studied metazoan genomes. Analysis for microProtein regulated protein classes revealed that transcription factors are a sizeable fraction of microProtein targets in human but only the exception in plants. This implies that regulation of protein activity by microProteins extends beyond the regulation of transcription factors and affects to a large extend other protein classes in plants.

### Outlook

The identification and experimental characterization of novel microProteins, based on miPFinder, will allow further improvement of the program. Knowledge of more microProteins will aid in refining the parameters in order to further improve the list of microProtein candidates. Additionally, future upgrades of the source databases will benefit microProtein identification. Most importantly, a complete and accurate annotation of all small transcripts and respective protein sequences including splice variants will allow for better microProtein detection.

In summary, selecting microProteins from miPFinder for experimental validation is ideally guided by taking all the above-mentioned criteria into account. For example, MIP1A- and MIP1B-related protein sequences are in majority large in size (65%), the relative fraction of microProteins is small (6%), and the sequence similarity is rated high. Additionally, MIP1A and MIP1B resemble an annotated protein–protein interaction domain (B-box) and MIP1A is annotated by STRING to interact with several large related proteins. They are also exclusively conserved among all three investigated dicots (Arabidopsis, tomato and potato). Therefore these candidates fit perfectly into the scheme of microProteins and were experimentally confirmed to have microProtein function (Graeff et al. 2016). However, when searching for microProteins with a specific function or protein category other priorities might be applicable.

Taken together, miPFinder allows the rapid identification of novel microProtein regulators and can be applied to any close-to-complete genome. All settings are adjustable thus allowing users to perform a variety of searches according to their needs. Up to date, microProteins are underinvestigated in animals compared with plants and miPFinder enables the identification of microProteins in all available genomes. The miPFinder algorithm is freely available under the GPLv3 license at https://github.com/DaStraub/miPFinder. 

## Supplementary Material

Supplementary data are available at *Genome Biology and Evolution* online.

## Supplementary Material

Supplementary Data
