# Supplementary material for: Cross-Species Genome-Wide Identification of Evolutionary Conserved MicroProteins
Source: Genome Biol Evol. 2017 Mar 1;9(3):777–89. doi: 10.1093/gbe/evx041 (PMC5381583; doi:10.1093/gbe/evx041)
Supplement: Supplementary Data [file evx041_Supp.zip › Suppl_Fig_text.docx]

**Supplementary figure S1:** MicroProtein alignment rating and e-value. The alignment rating favors known microProteins and is inversely related to BLAST/HMMER e-values.

**Supplementary figure S2:** Disease keywords (top 6) of human high probability microProtein candidates.

**Supplementary figure S3:** PANTHER protein classes that are regulated by Arabidopsis (left) and human (right) conserved microProteins.
