# Supplementary figures and images for: Cross-Species Genome-Wide Identification of Evolutionary Conserved MicroProteins
Source: Genome Biol Evol. 2017 Mar 1;9(3):777–89. doi: 10.1093/gbe/evx041 (PMC5381583; doi:10.1093/gbe/evx041)

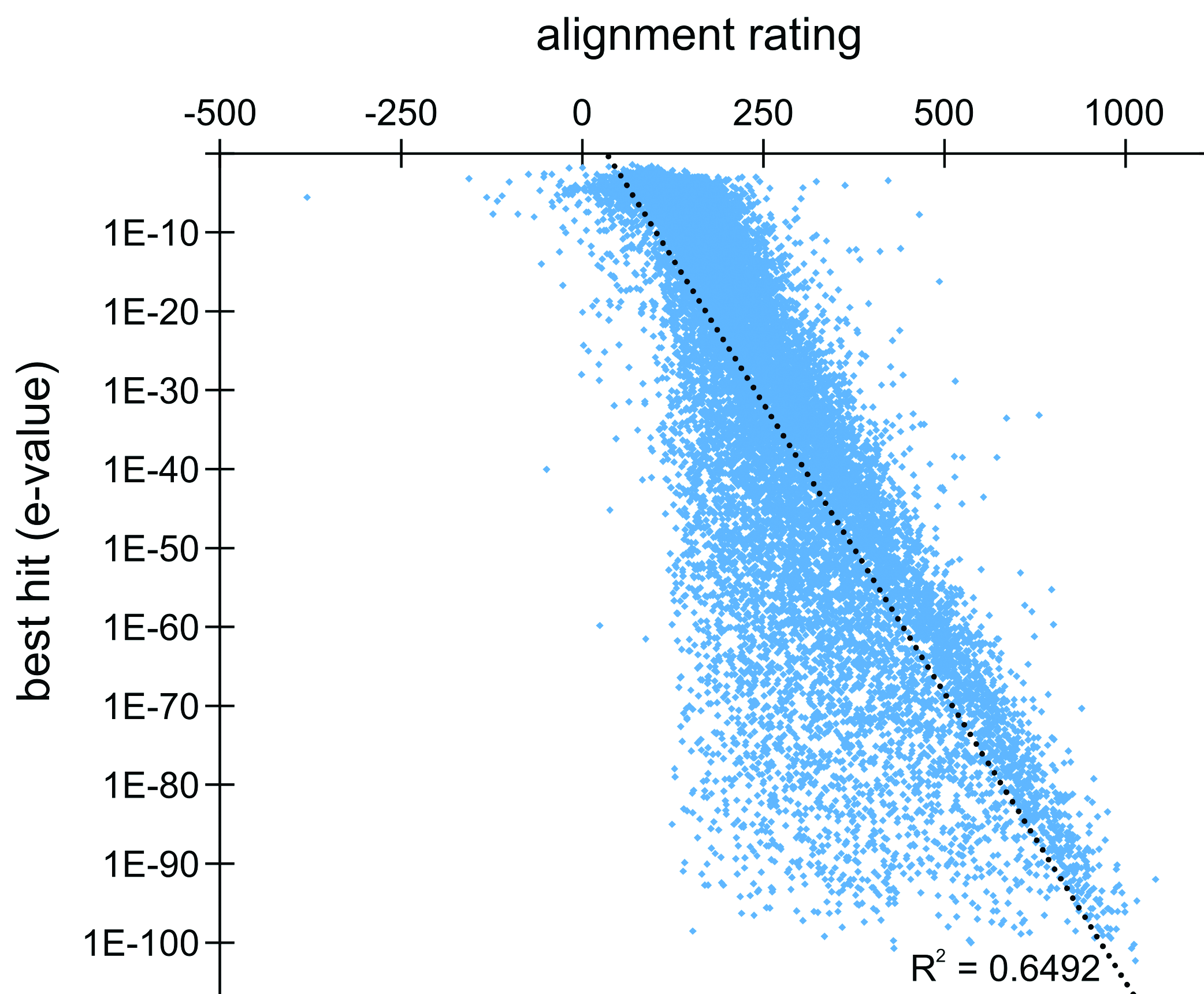

Supplement: Supplementary Data [file evx041_Supp.zip › Straub_SupplFig1.tif]

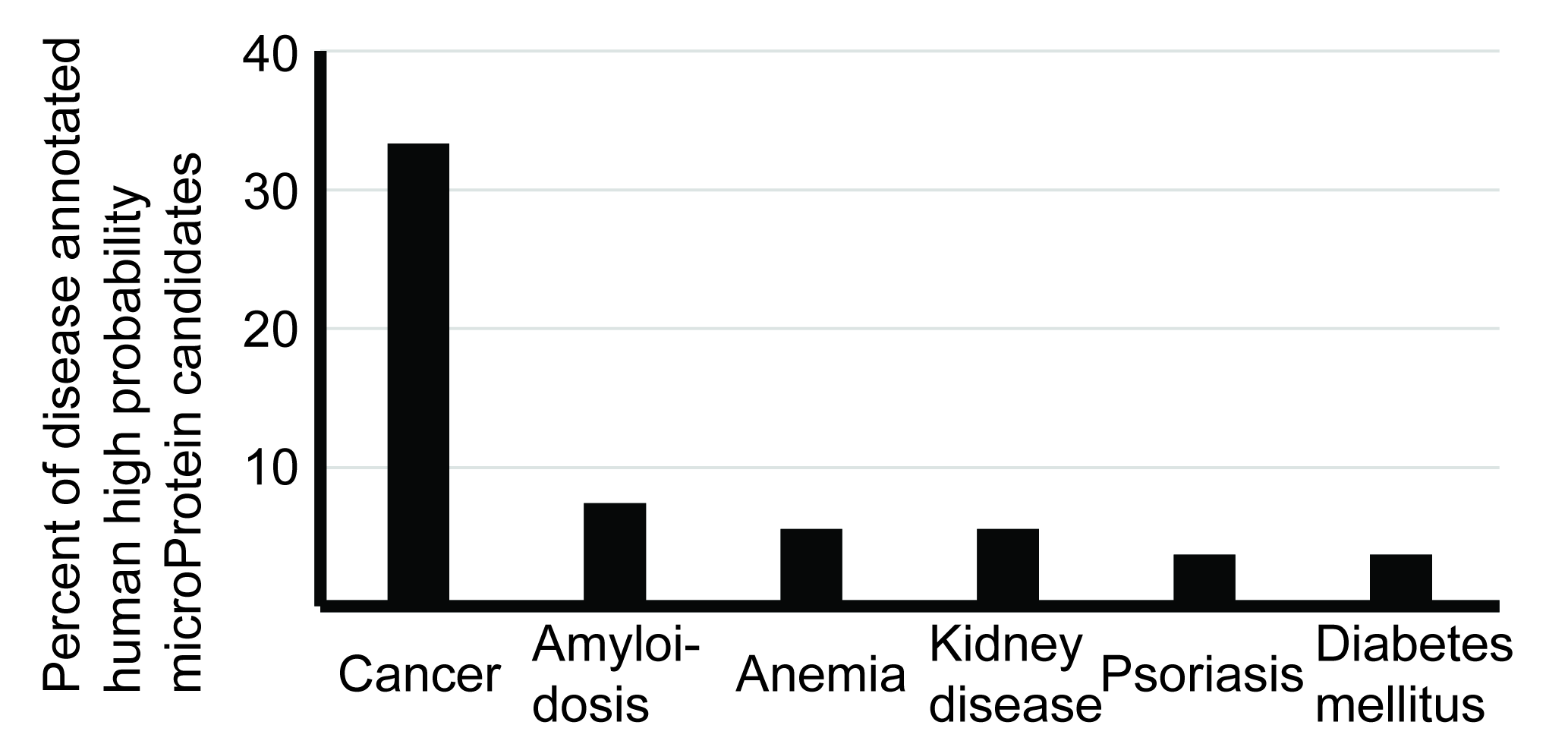

Supplement: Supplementary Data [file evx041_Supp.zip › Straub_SupplFig2.tif]

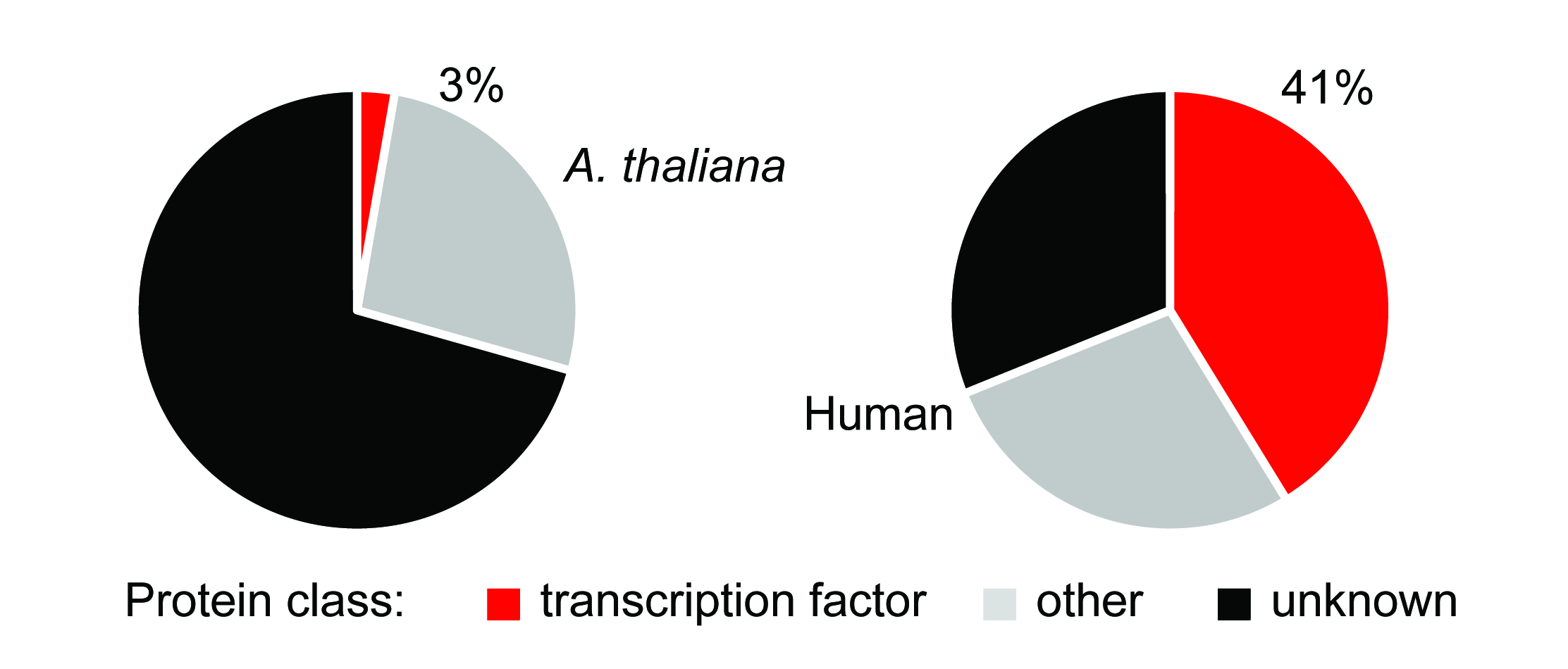

Supplement: Supplementary Data [file evx041_Supp.zip › Straub_SupplFig3.tif]
